# The Endophytic Fungi Diversity, Community Structure, and Ecological Function Prediction of *Sophora alopecuroides* in Ningxia, China

**DOI:** 10.3390/microorganisms10112099

**Published:** 2022-10-22

**Authors:** Ruotong Wang, Qingchen Zhang, Mingxiu Ju, Siyuan Yan, Qiangqiang Zhang, Peiwen Gu

**Affiliations:** 1 School of Agriculture, Ningxia University, Yinchuan 750021, China; 2 Department of Pharmacotherapy and Translational Research, University of Florida, Gainesville, FL 32611, USA

**Keywords:** *S. alopecuroides*, endophytic fungi, diversity, Illumina MiSeq sequencing, ecological function

## Abstract

*Sophora alopecuroides* L. has great medicinal and ecological value in northwestern China. The host and its microbiota are mutually symbiotic, collectively forming a holobiont, conferring beneficial effects to the plant. However, the analysis of diversity, mycobiota composition, and the ecological function of endophytic fungi in the holobiont of *S. alopecuroides* is relatively lacking. In this article, the fungal community profiling of roots, stems, leaves, and seeds of *S. alopecuroides* (at the fruit maturity stage) from Huamachi and Baofeng in Ningxia, China were investigated based on the ITS1 region, using high-throughput sequencing technology. As a result, a total of 751 operational taxonomic units (OTUs) were obtained and further classified into 9 phyla, 27 classes, 66 orders, 141 families, 245 genera, and 340 species. The roots had the highest fungal richness and diversity, while the stems had the highest evenness and pedigree diversity. There also was a significant difference in the richness of the endophytic fungal community between root and seed (*p* < 0.05). The organ was the main factor affecting the community structure of endophytic fungi in *S. alopecuroides*. The genera of unclassified Ascomycota, *Tricholoma*, *Apiotrichum*, *Alternaria*, and *Aspergillus* made up the vast majority of relative abundance, which were common in all four organs as well. The dominant and endemic genera and biomarkers of endophytic fungi in four organs of *S. alopecuroides* were different and exhibited organ specificity or tissue preference. The endophytic fungi of *S. alopecuroides* were mainly divided into 15 ecological function groups, among which saprotroph was absolutely dominant, followed by mixotrophic and pathotroph, and the symbiotroph was the least. With this study, we revealed the diversity and community structure and predicted the ecological function of the endophytic fungi of *S. alopecuroides*, which provided a theoretical reference for the further development and utilization of the endophytic fungi resources of *S. alopecuroides*.

## 1. Introduction

Endophytic fungi are defined as a class of fungi that live in plant tissues for all or a certain period of life and do not cause obvious disease symptoms [1,2]. The major species of endophytic fungi include saprophytes, latent pathogens, and mycorrhizal fungi [3,4]. Plants can be regarded as a holobiont containing host plants and their microbiota [5,6]. Specific endophytes may have evolved with their host as a holobiont, with potential pharmaceutical, agricultural, or environmental significance [7,8,9]. Studies have found that endophytic fungi colonize almost all plants with rich diversity, which are affected by host plant species, organs, geographical location, seasonal climate, and other factors [10]. The association between fungal endophytes and their host plant is mutualistic since they assist each other in several ways [1]. Plants provide the fungi with habitation and nourishment, whereas endophytic fungi in turn assist their host to cause positive impacts on host plant growth and development [1], improve the resistance of the host to biotic and abiotic stresses [11,12], and increase the synthesis and accumulation of secondary substances in medicinal plants [10]. As an important part of the plant holobiont, plant endophytic fungi are a current research hotspot for mining and utilizing beneficial microbial resources.

As a perennial herb of *Sophora* (Fabaceae), *S. alopecuroides* L. is widely distributed in the desert and semi-desert grassland in northwest China [13]. It shows favorable drought, salt, and alkaline resistance, anti-sandstorm performance, and strong nitrogen fixation, making it a vital pioneer in environmental protection [13]. The whole grass, roots, and seeds have different uses as medicine, with antitumor, antivirus, antimicrobial, and anti-inflammatory properties. *S. alopecuroides* is an important medicinal plant resource and natural vegetation component, its wild resources are widely distributed. To date, investigations on *S. alopecuroides* mainly focused on its morphology, ecology, and secondary metabolism, but studies on the endophytic fungi have been poorly researched [14]. Gu et al. [15] isolated 214 endophytic fungi from *S. alopecuroides* collected from five sample sites, belonging to 22 genera, of which *Oospora* and *Cephalosporium* were common genera among the five sample sites. Gao et al. [16] isolated 213 strains from 30 *S. alopecuroides* samples, belonging to 19 genera, of which *Fusarium*, *Alternaria*, and *Phoma* were the dominant genera. The endophytic fungi of *S. alopecuroides* have rich diversity. Most of the data on the diversity of endophytic fungi in plants are derived from studies relying on fungal culturing from surface-sterilized tissues. Evaluating microbial communities via the traditional culturing technique does not reveal the complete microbial diversity of endophytes because only 0.1–10% of microbes are culturable [17]. Thus, a comprehensive technique is urgently required for their annotation.

As a second-generation sequencing technology, high-throughput sequencing (HTS), realizes the direct sequencing of mass gene sequences [18]. Compared with traditional culturable methods, HTS can not only can comprehensively and objectively determine the microbial colony structure and the relative compositions of microbial communities in a target environment [19,20] but also help to study the diversity of symbiotic microbial ecological functions. Characterized by a fast sequencing speed, high throughput, and low operating cost, HTS provides a convenient and efficient method for fungal ecology research [21]. Currently, HTS has been widely used in soil microbial diversity [22], space microbiology [23], disease risk assessment [24], water environment monitoring [25], etc. In terms of endophytic fungi, most of them are to explore the community composition of plant endophytic fungi [17], the succession of dominant species [26], and the interaction between endophytic fungi and host [27]. In recent years, HTS has been applied to predict the diversity and function of symbiotic microorganisms in orchids [28], *Alpinia zerumbet seeds* [17], *Gentiana* [29], and other plants. Cui et al. [30] observed the diversity of the endophytic fungal community within *Huperzia serrata* by HTS, further detected the relevance to the production of HupA by the host plant, and confirmed that the HS7-1 strain could produce HupA. Liang et al. [31] revealed that the accumulation of pharmacological components in *Codonopsis pilosula* were related to the dynamic changes of endophytes, and further culture-dependent experiments validated that endophytes participated in the accumulation of metabolites. In the early stage, our research team used HTS to analyze the diversity of endophytic fungi of *S. alopecuroides* seeds in different geographical populations in China [32] and we hypothesized that endophytic fungi of *S. alopecuroides* in different organs are also diverse and have ecological functions. 

The microbial assembly of endophytic fungi is affected by the developmental stage of their host. Houlden et al. [33] confirmed that shifts in the diversity of fungal and bacterial communities were more pronounced in maturing pea and sugar beet plants. Li et al. [34] studied that the microbial community composition of the root system of *Lycium barbarum* were dynamic in response to the growth stage, and the diversity in the fruiting stage was the highest. Ajilogba et al. [35] found that the community structure of symbiotic microorganisms of the leguminous plant *Vigna subterranea* was different from that of other growth stages in the mature stage, while the community structure of other growth stages did not show significant differences. Our research used HTS for the first time to study the endophytic fungi of *S. alopecuroides* in the desert steppe of Ningxia, China. The main purposes are as follows: (1) to clarify the diversity of endophytic fungi in *S. alopecuroides*, (2) to analyze the changes in the composition and diversity of endophytic fungi in different organs of *S. alopecuroides*, and (3) to predict the function of endophytic fungi in *S. alopecuroides*. This study provides a theoretical basis for the development and utilization of endophytic fungi resources of *S. alopecuroides* in the arid desert area of Ningxia, China.

## 2. Materials and Methods

### 2.1. Research District’s General Situation

Huamachi of Yanchi County (38°28′40″ N, 106°38′11″ E, 1212.94 m) and Baofeng Energy Base of Ningdong (37°47′43″ N, 107°31′3″ E, 1336.40 m) are located in the Ningxia Baijitan National Nature Reserve. This area is located at the southwest edge of the Mu Us Desert in China and is a typical arid desert grassland. The southern part of the area is mainly located among sandy hills and the northern part is mainly characterized by mountainous deserts. This area belongs to the arid climate zone of the middle temperate zone, with significant continental climate characteristics. The annual average temperature is 8.6 °C, the annual average rainfall is 198.9 mm, and the annual average evaporation of 1928.4 mm. The vegetation type is dominated by psammophytes, which are suitable for living in deserts and semi-deserts due to their high tolerance to drought and salinity. The dominant plant taxa include: Fabaceae, such as *S. alopecuroide*, *Glycyrrhiza uralensis*, *Oxytropis aciphylla*, *Caragana korshinskii*, *Caragana tibetica*, *and Ammopiptanthus mongolicus*; Poaceae, such as *Pennisetum centrasiaticum*, *Agropyron desertorum*, *Achnatherum splendens*, and *Stipa breviflora*; Zygophyllaceae, such as *Peganum nigellastrum*; Asteraceae, such as *Artemisia desertorum*; Iridaceae, such as *Irisbungei*, etc.

### 2.2. Samples Collection

In northwestern China, *S. alopecuroides* germinates in mid-April, its flowering period is from May to June, its fruiting period is from August to October, and the growth period is in the range of 140–150 days [14]. For the study, healthy plants of wild *S. alopecuroides* in the fruit maturity stage were collected from two sites in Huamachi (H) and Baofeng Energy Base (B), Ningxia Yanchi County, Ningxia, China on 1 September 2021. At each site, three sampling points (10 m × 10 m) were set up by the diagonal sampling method, and the distance between each point was more than 150 m. After removing 20 cm of dead leaves and debris around the *S. alopecuroide*, we selected healthy, disease- and pest-free, and mechanically damage-free *S. alopecuroides* plants, excavated *S. alopecuroides* plants about 40 cm vertically downward, and the soil was mulched after sampling for lessening the damage to the ecological environment. A total of 10 plants were collected (sampling distance of 10 m) at each sampling point. The samples were placed in sterile fresh bags, marked and stored at low temperature, and brought back to the laboratory immediately. We separated the root (R), stem (St), leaf (L), and seed (S) tissues with sterile scissors, mixed the same organs of 10 *S. alopecuroides* plants at each sampling point as one sample, and retained 5 g each using the quartering method. There were 24 samples in total for sample pretreatment within 24 h.

### 2.3. Sample Pretreatment

Samples of fresh, healthy *S. alopecuroide* plants were collected and repeatedly rinsed under running water to remove soil and appendages from the sample surface and sterilized with pure culture [36]. The sterilization procedures were as follows: the *S. alopecuroide* organ samples were rinsed 5 times with sterile water and then the surface bubbles were eliminated with 75% alcohol for 30 s. The surface sterilization was then performed with 1% sodium hypochlorite (m/v) for 0.5~4 min according to organ size (generally, 4 min for roots, 2 min for stems, 0.5 min for leaves, and 3 min for seeds), and with sterile water organ samples were rinsed 3 times. Plants were placed on sterile filter paper for drying and wrapped with sterile tin foil, labeled, and stored at −80 °C for high-throughput sequencing of *S. alopecuroide* endophytic fungi. To confirm the successful disinfection of the seed surface, the final water rinse was spread onto a potato dextrose agar (PDA) plate and cultured for 3 d in the dark at 25 °C. If no colonies were observed, the samples were assumed to be adequately disinfected and were used for DNA extraction, amplification of the ITS1 region of rDNA, and high-throughput sequencing.

### 2.4. DNA Extraction, Amplicon of ITS rDNA Region, and High-Throughput Sequencing

Total *S. alopecuroides* DNA was extracted using a DNA extraction kit (Fast DNA^®^ SPIN Kit, MPBIO, Santa Ana, USA) and DNA amplification experiments were performed using an ABI GeneAmp^®^ Model 9700 PCR instrument. Fungal primers ITS1F (5’-CTTGGTCATTTAGAGGAAGTAA-3’) and ITS2R (5’-GCTGCGTTCTTCATCGATGC-3’) were used to amplify the ITS1 region of rDNA [37]. The reaction system was 20 μL: 10×Buffer 2 μL, 2.5 mmol/L dNTPs 2 μL, 5 μmol/L 0.8 μL each for forward and reverse primers, TaKaRa rTaq DNA Polymerase 0.2 μL, BSA 0.2 μL, DNA template 10 ng, and ddH_2_O was made up to 20 μL. PCR procedure: 95 °C for 3 min; 95 °C for 30 s, 55 °C for 30 s, 72 °C for 45 s; 72 °C for 10 min, 10 °C for holding. PCR products were sampled on 2% agar gel electrophoresis in 3 μL for detection.

The purity and concentration of PCR products and DNA integrity, PCR amplification, and Illumina MiSeq sequencing were performed by Majorbio Co., Ltd. (Shanghai, China). All sequencing data were deposited to the NCBI Sequence Read Archive2 under project number PRJNA832900. 

### 2.5. Processing of Sequencing Data

The paired-end reads from sequencing were assigned to respective samples using Cutadapt software [38] based on their specificity. Barcode and primer sequences were truncated from these reads [32]. To avoid mixing samples, specific primer-based barcodes were assigned to each sample at the start and end of sequencing [39]. In HTS-based studies, samples are usually amplified with primer variants equipped with unique identifier indices (also known as tags, molecular identifiers, or barcodes) to recognize the samples bioinformatically [40]. These indices should be longer than six bases and differ from each other by at least three nucleotides to prevent technical cross-contamination; unique indices should be used for each sample on both the forward and the reverse primer to recognize index-switching artefacts [40]. Flash software version 1.2.7 [41] was applied to cut and splice the remaining paired-end reads of each sample, and the paired-end clean readout was merged into the original label. High-quality clean data were obtained by filtering the original data under specific filtering conditions [42,43]. Additionally, to segregate chimeric sequences and eliminate the nonmicrobial reads, the reads were compared with the Unite database (https://unite.ut.ee/, accessed on 5 November 2021.) [44] using UCHIME [45] to generate. UPARSE software version 7.0.1001 (http://drive5.com/uparse/, accessed on 13 November 2021) [46] was used to cluster the sequences into the same operational taxonomic units (OTUs) with ≥97% similarity, and a representative sequence with the highest frequency was selected for further annotation. The Unite database (Release 8.0 http://unite.ut.ee/index.php, accessed on 23 November 2021) was used to execute annotated information for each representative sequence. OTU abundance was normalized using the samples containing the least sequences (35,140 bp). Subsequent analyses were based on the normalized data.

### 2.6. Statistical Analysis

The alpha diversity indexes [47] were calculated by Mothur software (version v.1.30.2). Duncan’s multiple range test was used for the intergroup test of the alpha diversity indexes in four organs, and Student’s *t*-test was used for the intergroup test of the alpha diversity indexes in two sites (IBM SPSS Statistics 26). The normalized OTU table was analyzed using Bray–Curtis metrics and utilized to evaluate the β diversity and to construct PCoA plots [48] using R software (version 3.3.1) [49] on the Majorbio I-Sanger Cloud Platform. In order to compare fungal community composition and to partition off variance in different organs, Bray–Curtis distance matrices were subjected to PERMANOVA [50] using the adonis function with a permutation number of 999, available in the vegan package of R. Taxonomic compositions were visualized based on relative abundances using R software (version 3.3.1) on the Majorbio I-Sanger Cloud Platform. The number of common and unique genera in each sample was counted and a Venn diagram was constructed [17] based on R software (version 3.3.1) on the Majorbio I-Sanger Cloud Platform. The linear discriminant analysis effect size (LEfSe, http://huttenhower.sph.harvard.edu/galaxy/root?tool_id=lefse_upload, accessed on 29 March 2022) algorithm was used to identify taxa (genus level) that differed in relative abundance between the organs. Taxa with LDA scores higher than two were identified as biomarkers in LEfSe [51]. The FUNGuild [52] database (http://www.funguild.org/, accessed on 25 May 2022) was used to predict the ecological function categories of fungi. The above microbiological data were analyzed on the free online platform Majorbio I-Sanger Cloud Platform (http://www.i-sanger.com, accessed on 10 January 2022).

## 3. Results

### 3.1. Overview of the Sequencing Data of Fungal Communities

**Figure 1:**
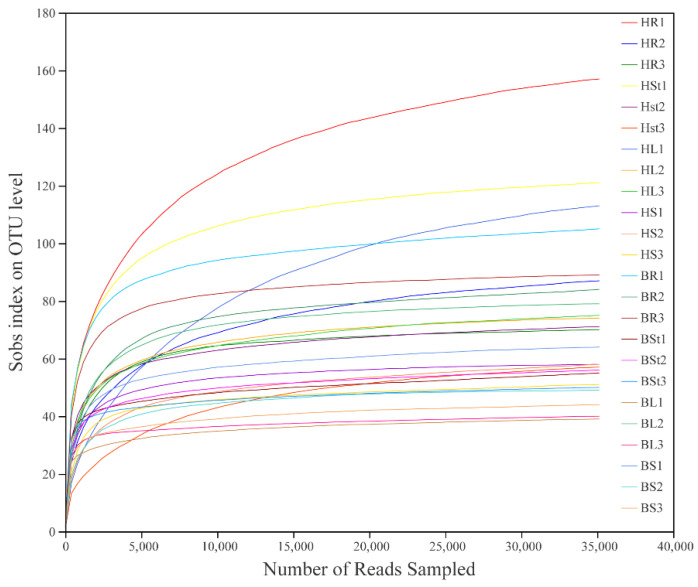
Rarefaction curve of endophytic fungi in 24 *S. alopecuroides* samples based on sobs index. Note: the abscissa represents the amount of randomly selected sequencing data; the ordinate represents the number of species observed.

A total of 24 samples of *S. alopecuroides* roots, stems, leaves, and seeds were sequenced in high throughput and a total of 133,916 high-quality sequences with an average sequence length of 246 bp were obtained after quality filtering. At a 97% similarity level, a total of 751 operational taxonomic units (OTUs) were obtained. Moreover, the fungal OTUs were assigned to 9 phyla, 27 classes, 66 orders, 141 families, 245 genera, and 340 species. The rarefaction curve reflects the sequencing depth of the sample, which can be used to assess whether the sequencing volume is sufficient to cover all taxa. As shown in Figure 1, the rarefaction curve indicated that the number of OTUs was sufficient and saturated in each sample.

### 3.2. Alpha Diversity of Endophytic Fungi of S. alopecuroides

The alpha diversity of the endophytic fungi of *S. alopecuroides* in the 24 samples was assessed by the sequence number of samples, community diversity (Simpson index), richness (Ace index), evenness (Shannoneven index), and pedigree diversity of species (PD index) (Table 1, Table S1). The sequence number and Ace index between the two sites showed H > B, and the Simpson index, Shannoneven index, and PD index showed B > H, indicating that the community diversity, evenness, and pedigree diversity of endophytic fungi of *S. alopecuroides* in Huamachi were lower than those in Baofeng, but the fungal richness was significantly higher (*p* < 0.05). The highest sequence number detected among the four organs was for leaf, the Ace index showed the highest for root, the Simpson index showed the lowest for root, and the Shannoneven and PD indexes showed the highest for stem. That means the root had the highest richness and diversity, the stem had the highest evenness and pedigree diversity, and there was a significant difference in the richness between root and seed (*p* < 0.05). The above results indicate that the diversity of endophytic fungi of *S. alopecuroides* varies widely in different regions and organs.

### 3.3. Beta Diversity of Endophytic Fungi of S. alopecuroides

**Figure 2:**
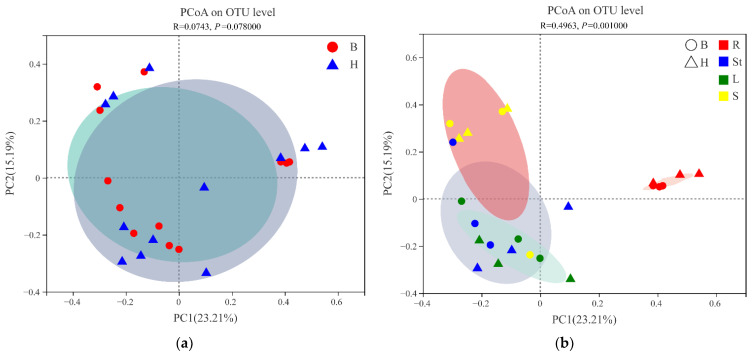
Principal coordinates analysis (PCoA) based on the relative abundance of fungal OTUs showing the fungal community structure: (**a**) in different sample sites: B (Circular), H (triangle); (**b**) in four organs: R (red color), St (blue color), L (green color), S (yellow colors).

The beta diversity of *S. alopecuroides* fungal communities was assessed at the OTU level to compare the similarities and differences in sample sites and organs. Principal coordinate analysis (PCoA) based on the ANOSIM inter-group difference test by the Bray–Curtis distance algorithm showed that PC1 and PC2 explained 23.21% and 15.19% of the total variation in endophytic fungi, respectively, with a total explanation of 38.4% (Figure 2). The fungal communities of the two sample sites overlapped to a high degree (Figure 2a) and the structure of the endophytic fungal community of *S. alopecuroides* did not differ significantly between the sample sites (R = 0.0743, *p* = 0.0780). The four organs formed individual clusters (Figure 2b), and the endophytic fungal community structure (R = 0.0963, *p* = 0.0010) was significantly different. The endophytic fungal communities in the roots and seeds were the most distant from each other, indicating the greatest difference between the two mycota structures, while the endophytic fungal communities in the stems and leaves were obviously clustered, indicating a smaller difference between the two (Figure 2b). These results were confirmed by adonis pairwise comparisons of microbiota structure between *S. alopecuroides* sites and organs (Table 2). A PERMANOVA test (Table 3) revealed that different organs better explained (0.33) and had a higher confidence level for (0.001) the variation in *S. alopecuroides* fungal communities, while sample sites poorly explained (0.07) and had a lower confidence level for (0.06) the variation in samples. That confirmed the significant differences in community structures among the organs (*p* < 0.05). As a result, plant organ was an influential factor in explaining the variation of *S. alopecuroides* fungal communities.

### 3.4. Fungal Composition and Relative Abundance among Different Organs of S. alopecuroides

**Figure 3:**
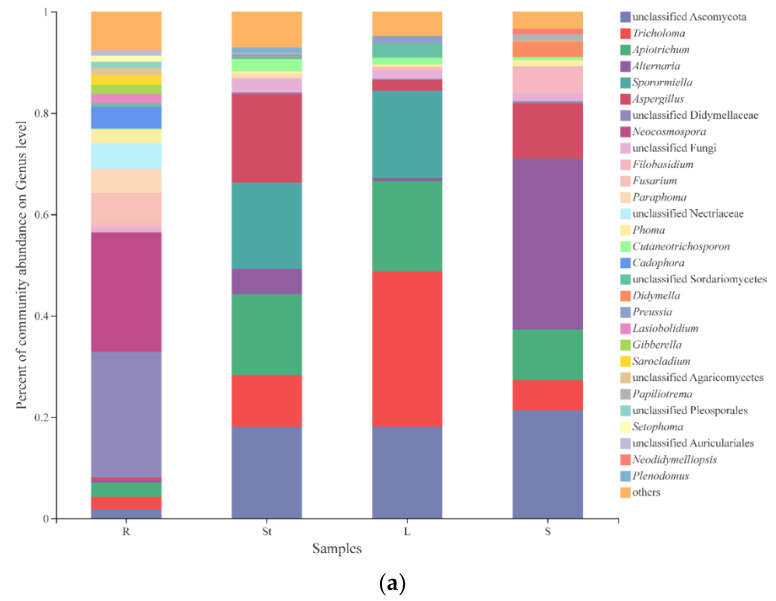

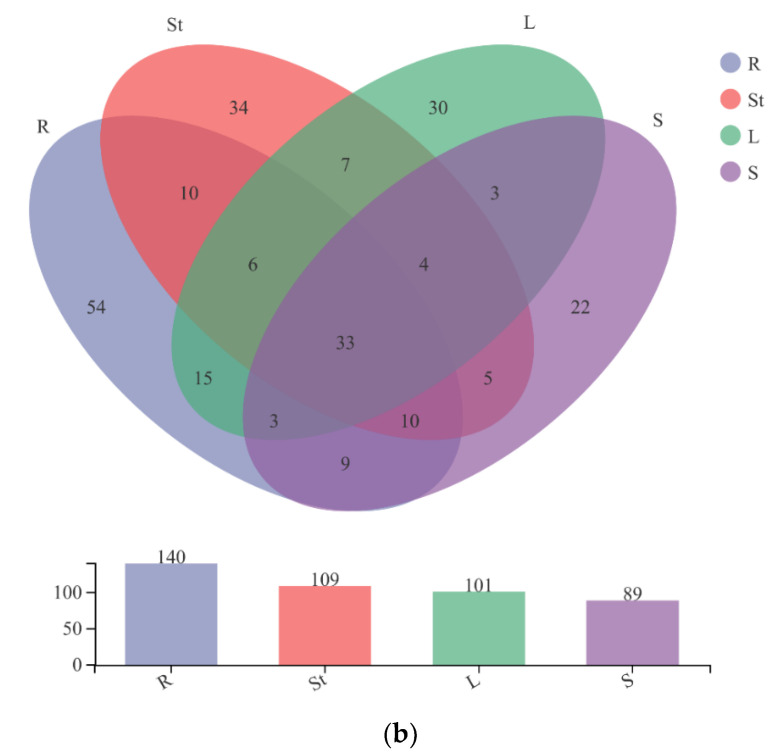
Fungal composition and relative abundance of *S. alopecuroides*: (**a**) different organs’ community composition of *S. alopecuroides* at genus level; (**b**) Venn diagram showing shared and unique fungal genera in each organ (root, stem, leaf, and seed).

It was confirmed that plant organ was the most influential factor to explain the variation of *S. alopecuroides* fungal community by the above analysis, so our subsequent analysis was mainly focused on four organs. We totally detected 9 phyla, 27 classes, 66 orders, 141 families, 245 genera, and 340 species of endophytic fungi in *S. alopecuroides*. At the genus level, excluding unidentified and relatively small (<0.01) fungal species, the six genera with the highest relative abundance were unclassified Ascomycota (14.82%), *Tricholoma* (12.33%), *Apiotrichum* (11.64%), *Alternaria* (9.94%), *Sporormiella* (8.56%), and *Aspergillus* (7.77%) (Figure S1). As shown in Figure 3a, there were significant differences in the endophytic fungal community composition and relative abundance between different organs. The diversity of endophytic fungi in roots was the highest, and the dominant genus was unclassified Didymellaceae with a relative abundance of 24.72%, followed by *Neocosmospora* (23.63%). The dominant genus in the stems was unclassified Ascomycota with a relative abundance of 18.07%, followed by *Aspergillus* (17.46%), *Sporormiella* (17.00%), *Apiotrichum* (15.94%), and *Tricholoma* (10.20%). The dominant genus in the leaves was *Tricholoma* with a relative abundance of 30.69%, followed by unclassified Ascomycota (18.08%), *Apiotrichum* (17.82%), and *Sporormiella* (17.21%). *Alternaria* was the dominant genus in the seeds with a relative abundance of 33.67%, followed by unclassified Ascomycota (21.34%) and *Aspergillus* (10.93%).

The Venn diagram (Figure 3b) visualizes the composition of shared and unique species shared by different organ *S. alopecuroides* samples at the genus level, with a degree of both intersection and independence. As can be seen from Table 4, the six genera with the highest relative abundance were unclassified Ascomycota (18.42%), *Tricholoma* (15.31%), *Apiotrichum* (14.47%), *Alternaria* (12.35%), *Aspergillus* (9.65%), and *Didymellaceae* (7.96%), of the 33 shared genera in four organs. The fungal composition of the four organs varied. The roots had 140 genera, with 54 unique genera, of which *Cadophora* (50.41%) had the highest abundance. The stems had 109 genera, with 34 unique genera, of which *Plenodomus* (38.25%) had the highest abundance. The leaves had 101 genera, with 30 unique genera, of which *Dioszegia* (23.43%) had the highest abundance. The seeds had 89 genera, with 22 unique genera, of which *Papiliotrema* (69.88%) had the highest abundance. The number of unique genera showed a decreasing trend by root, stem, leaf, and seed (54 > 34 > 30 > 22), and whether these unique genera affect the growth, development, and metabolism of the host plant needs further study.

### 3.5. Analysis of Endophytic Fungal Biomarkers in Different Organs of S. alopecuroides

**Figure 4:**
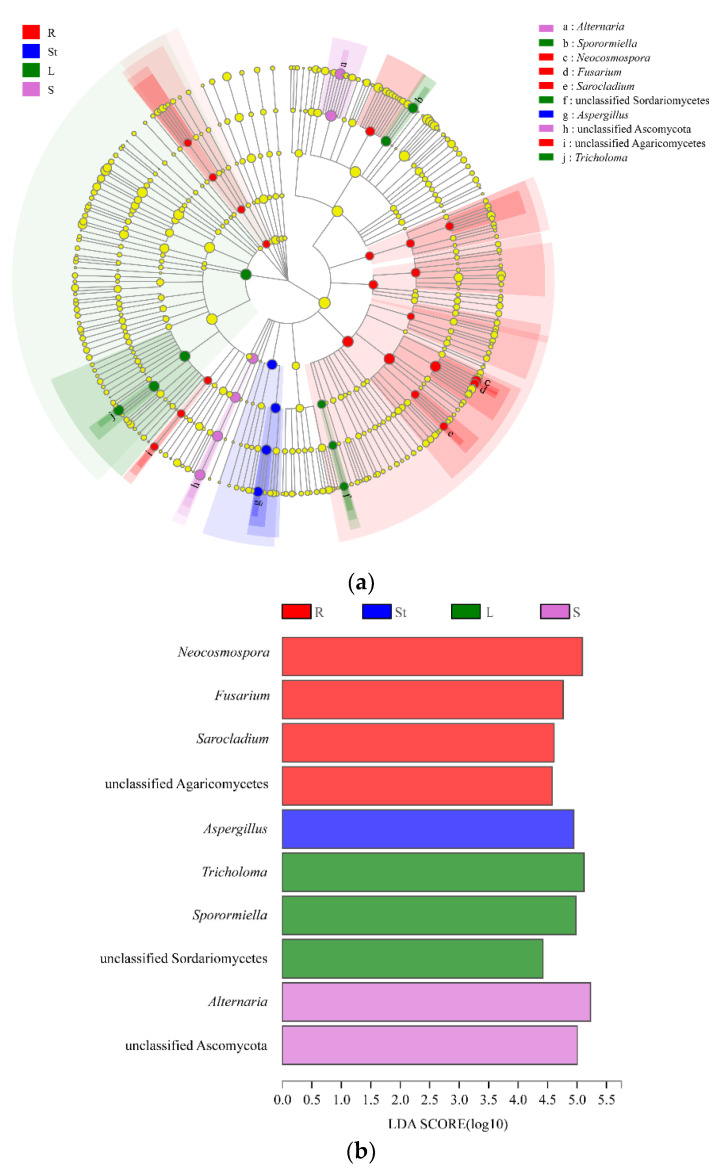
Biomarkers in different organs of *S. alopecuroides*: (**a**) LEfSe was used to identify the most differentially abundant taxa among four organs of *S. alopecuroides*. Cladogram generated by LEfSe indicating differences between the four organs at phylum, class, order, family, and genus levels. Each successive circle represents a phylogenetic level. Color regions indicate taxa enriched in the different organs. Differing taxa are listed on the right side of the cladogram. (**b**) Bar graph showing LDA scores for fungal taxa; only taxa meeting an LDA significant threshold >2 are shown.

The LEfSe analysis [53] was used to find biomarkers, which are statistically different at the genus level in the endophytic fungi of different *S. alopecuroides* organs. LEfSe analysis demonstrated (Figure 4a) that the *Neocosmospora*, *Fusarium*, *Sarocladium*, and unclassified Agaricomycetes were significantly enriched in roots. *Aspergillus* was significantly enriched in stems. *Tricholoma*, *Sporormiella*, and unclassified Sordariomycetes were significantly enriched in leaves. Additionally, *Alternaria* and unclassified Ascomycota were significantly enriched in seeds. LDA analysis (LDA > 2) showed (Figure 4b) that *Neocosmospora*, *Aspergillus*, *Tricholoma*, and *Alternaria* had the greatest effect on the four *S. alopecuroides* organs: roots, stems, leaves, and seeds, respectively.

### 3.6. Functional Guild Function Prediction of Endophytic Fungi in Different Organs of S. alopecuroides

**Figure 5:**
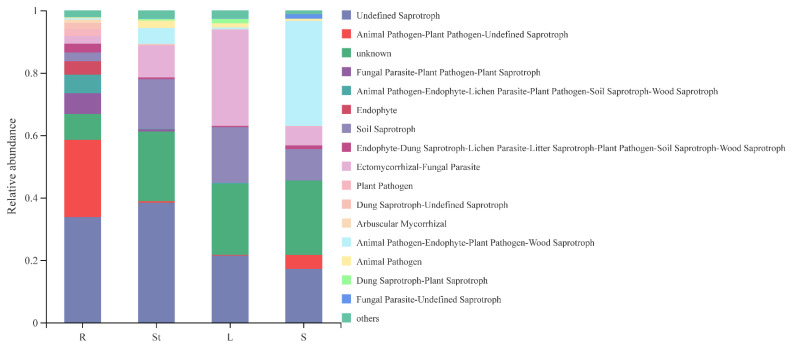
Ecological function analysis of endophytic fungi in various organs of *S. alopecuroides*.

Functional Guild (FUNGuild) is a tool used for the classification and analysis of fungal communities in a microecological guild [52]. The results of the FunGuild software-based analysis showing the combined species abundance <0.01 and the unknown functional groups, a total of 15 major ecological functional groups of *S. alopecuroides* were annotated (Figure 5). Among the 15 ecological functional groups of endophytic fungi of *S. alopecuroides*, 4 were saprotroph, including undefined Saprotroph, soil saprotroph, dung saprotroph-plant saprotroph, and dung saprotroph-undefined saprotroph, totaling 40.39%. Only two were pathotroph, including plant pathogen and animal pathogen, which accounted for 1.93%, and two were symbiotroph, including endophyte and arbuscular mycorrhizal with 1.41%. Moreover, there were seven mixed trophic types of fungi: ectomycorrhizal-fungal parasite, animal pathogen-endophyte-plant pathogen-wood saprotroph, animal pathogen-plant pathogen-undefined saprotroph, fungal parasite-plant pathogen-plant saprotroph, animal pathogen-endophyte-lichen parasite-plant pathogen-soil saprotroph-wood saprotroph, endophyte-dung saprotroph-lichen parasite-litter saprotroph-plant pathogen-soil saprotroph-wood saprotroph, fungal parasite-undefined saprotroph, totaling 34.91%. In addition, a large proportion (19.25%) of the flora with unknown functions were also present and their functions need to be further explored.

## 4. Discussion

Along with the development and maturation of the new generation sequencing technology, the analysis of microbial community structure and diversity is gradually developing from the with-culture method to the culture-free method and from a low-resolution level to a high-resolution level. In this study, the community structure and species richness of endophytic fungi of *S. alopecuroides* in Ningxia, China were analyzed for the first time with HTS. The results of our study demonstrated that the endophytic fungi of *S. alopecuroides* included 9 phyla, 27 classes, 66 orders, 141 families, 245 genera, and 340 species. Among the dominant genera, the result of *Alternaria* was identical to the previous culturable method [15,16,54], while *Tricholoma*, *Apiotrichum, Sporormiella,* and *Aspergillus* were reported for the first time. The fungal composition obtained by the traditional cultural method and HTS is not completely consistent with previous reports [19,20]. The reason for this difference may be due to the varying methods used to study the diversity of endophytic fungal communities. Owing to the limitations of medium composition and culture conditions, the with-culture method preferentially selects some species in the sample and the species that grow rapidly in culture overwhelm the species that grow slowly, so fewer taxa are obtained [55]. However, the HTS method was able to make up for the selection limitations of traditional culture selection, realize the in situ analysis of microbial population structure in a natural state, and present the microbial community structure information that is closest to the real state in plants [56]. In most cases, the taxon obtained by HTS is higher than the culturable method and has more accuracy and reliability.

The differences in the composition of plant endophytic microbial communities depend not only on plant species, living environment, different stages of plant growth and development, and health status but also on plant organs or tissues that play an important role in microbial colonization [57,58]. Siddique et al. [49] reported on the microbial communities of buds and twigs in two beech forests using HTS and found that it was plant organs rather than the habitat that determined the endogenous microbiota of young beech trees. Küngas et al. [59] compared the diversity and community structure of fungi inhabiting leaves, branches, and trunks of *Alnus incana* and *Corylus avellana* growing at three hemiboreal forest sites. The results showed that tree organs were the main determinants of fungal community structure, whereas the effects of the host species and locality remained secondary and negligible. Among them, the fungal community structure in the trunk was significantly different from that in the leaves and branches. We also found that different organs are the main reason for the differences in the community structure of endophytic fungi in *S. alopecuroides*, which may be related to the fact that the endophytic fungi are in an in planta environment and the fungal community is less affected by the external environment.

All types of plant tissues can accommodate endophytic fungal communities [17], and fungal communities in different plant organ hosts have certain organizational preferences [60]. Sun et al. [61] conducted cluster analysis and Sorenson’s similarity coefficient analysis on the endophytic fungi isolated from the leaves and branches of six medicinal plants, and the results showed that endophytic fungi have a certain degree of host and tissue preference. Su et al. [62] found that the colonization rate of endophytic fungi in the roots of *Stipa grandis* in the Inner Mongolia steppe was significantly higher than that in leaves and the composition and dominant population of endophytic fungi in roots and leaves were also completely different. Similarly, the dominant and unique genera and biomarkers of endophytic fungi in various organs of *S. alopecuroides* showed obvious tissue specificity. This mainly results from the differences in the internal environment of different tissues and organs (such as oxygen content, enzymes, chemical components, nutrients, etc.), forming a unique microenvironment and resulting in a specific selection of survival sites by endophytic fungal communities [20].

It is worth noticing that the roots had the highest diversity and richness of endophytic fungal communities in *S. alopecuroides*, while the seeds and leaves are the lowest. Ren et al. [20] analyzed the diversity of flora communities in different organs of the Pinggu peach and found that the sobs index and lnvsimpson index showed root > flower > stem > leaf, and the diversity and richness of endophytic fungi in roots were the highest. Chen et al. [63] further analyzed the high diversity of root endophytic fungi, which may be due to the fact that there are more litter residues (fallen leaves, branches, and dead roots) and rhizosphere exudates at the contact interface between roots and soil. They can provide sufficient available carbon, nitrogen, and trace elements for rhizosphere and root fungi, which in turn promote the improvement of their richness. In leaves, microorganisms first colonize the leaf surface of plants through stomata, water holes, wounds, etc., through horizontal transmission and then enter the leaf tissue. Due to ultraviolet radiation, lack of nutrients, and drying, the aboveground part of the plant has a poor nutritional environment, resulting in the reduction of the number of microorganisms in the leaves [3]. Hardoim et al. [3] supported that endophytic fungi spread vertically through seeds, which are highly protected closed organs of plants, resulting in fewer species and numbers of endophytic fungi than other organs [64]. Ecological function groups are divided based on trophic types, which refer to a group of related or unrelated species that use the same type of ecological resources in a similar way [65]. The functional group categories, relative numbers, and annotated taxa at different levels actually involved in each sample represent the ecological functional structure of the fungal community in the sample. We here found that the saprotroph was the major type in the endophytic fungal community of *S. alopecuroides*, followed by the mixed trophic and pathotroph, and the symbiotroph was the least. This is consistent with the results of Zhou et al. [66] and Zhou et al. [67], which showed that saprotroph is the dominant type of endophytic fungi in *Cymbidium ensifolium* and tobacco roots. However, this is different from the general recognition that most plant endophytes are saprotroph with plants. Of course, FunGuild predicted the biological function of fungi based on the known references, while the biological function of the same fungus to different hosts in different environments is different [66]. Saprophytic fungi and animal feces in the soil carry more animal pathogenic fungi, which can enter and colonize plant roots through root hairs, lenticels, cracks in lateral roots, etc., forming endophytic fungi and functional groups [67]. In addition, the functional groups of the endophytic composition in *S. alopecuroides* are a combination of mixed trophic types rather than a single ecological type, such as ectomycorrhizal-fungal parasite, animal pathogen-endophyte-plant pathogen-wood saprotroph, animal pathogen-endophyte-lichen parasite-plant pathogen-soil saprotroph-wood saprotroph, endophyte-dung saprotroph-lichen parasite-litter saprotroph-plant pathogen-soil saprotroph-wood saprotroph, and other ecological types. Endophytic fungi play different roles in different life cycles of plants and are at an unstable stage in development. They are endophytic fungi when plant tissues grow healthily, but they become saprophytic fungi when tissues age or die. This may be due to the fact that endophytic fungi choose the corresponding survival strategy to adapt to complex and volatile living conditions [68]. There are a small number of animal pathogens in the endophytic community of *S. alopecuroides*. Peter et al. [69] explained that some pathogenic microorganisms can infect different biological hosts and infect humans and animals across borders in nature. In the endophytic fungi of *S. alopecuroides*, the symbiotroph accounts for the smallest proportion. With the increase in the amount of this trophic type, the pathotroph fungi and saprotroph fungi are affected at the same time. On the one hand, symbiotroph fungi can antagonize pathogenic fungi, reduce the colonization of pathogenic fungi, and protect plants from disease infection [70], resulting in the continuous reduction of pathotroph fungi. On the other hand, mycorrhizal fungi, especially ectomycorrhizal fungi, ingest a large number of nutrients in the soil, causing the growth of saprophytic fungi and other microbial decomposers to be inhibited [71]. Therefore, when symbiotic trophic fungi are dominant, the host’s anti-interference ability increases [72], while the proportion of pathotroph fungi and saprophytic fungi is higher than that of symbiotic trophic fungi, it is very easy to cause plant diseases [73]. We also acknowledge that while FUNGuild is useful for fungal functional group assignment [74], many OTUs were placed into the unassigned group, as the FUNGuild database is currently incomplete [52].

In *S. alopecuroides*, we detected some valuable endophytic fungi, which provided useful information for further research on the specific ecological functions of endophytic fungi in *S. alopecuroides*. Unclassified Ascomycota, *Alternaria*, and *Aspergillus* are the dominant genera in *S. alopecuroides*, which were common in all four organs as well. It is reported that plant fungi are mainly composed of Ascomycota [20,32], which is consistent with the result that unclassified Ascomycota is the dominant genera in this study. Ascomycota fungi have higher species diversity due to their faster evolutionary rate and adaptability [75]. *Aspergillus* plays an active role in the field of plant biological control. Elkady et al. [76] supported that the active metabolites in the endophytic fungus *Aspergillus niger* OL519514 isolated from *Opuntia ficus-indica* were potential bioactive antimicrobial compounds against multidrug-resistant bacteria. Morales-Sánchez et al. [77] confirmed that an ethyl acetate extract from the endophytic fungus *Aspergillus* sp SPH2 isolated from the stem parts of the endemic plant *Bethencourtia palmensis* was screened for its biocontrol properties against plant pathogens. Seven metabolites of *Aspergillus* sp. KJ-9 have inhibitory activities against *Gibberella saubinetti*, *Magnaporthe grisea*, *Botrytis cinerea*, *Colletotrichum gloeosporioides*, and *Alternaria solani* [78]. *Aspergillus* can also promote plant growth and maintain the health of host plants. The *Aspergillus fumigatus* sp. LH02 separated from the soybean roots can significantly promote the shoot length, shoot fresh and dry biomass, leaf area, chlorophyll contents, and photosynthetic rate of soybean plants [79]. Indole-3-acetic acid (IAA) in the secondary metabolite of *Aspergillus* awamori Wl1 could enhance the growth of the host plant [80]. In addition, fumimycin produced by *Aspergillus* is a target in antibacterial, antimalarial, and anticancer drug discovery [81]. *Alternaria* is a ubiquitous fungal genus that includes saprobic, endophytic, and pathogenic species [82]. *Alternaria* can produce structurally unique and bioactive substances, such as isobenzofuranone A and indandione B, which showed significant inhibitory activities against four tumor cell lines and α-glucosidase [83]. Likewise, *Alternaria* has great potential in the biological control of plant diseases [84]. Ju et al. [32] found that specific endophytic fungi play a key role in the accumulation of bioactive compounds in the internal environment of *S. alopecuroides* seeds, and *Alternaria* in *S. alopecuroides* can promote the production of quinolizidine alkaloids (QAs) in the host. Endophytic fungi of *S. alopecuroides* have rich diversity. At present, studies have proved that they have antimicrobial activity [15] and function of promoting host alkaloid synthesis [32,85].

Our research provides a theoretical basis for the development and utilization of the endophytic fungi resources of *S. alopecuroides*. In the future, further research can be carried out in the following aspects: (1) the culture medium for separating certain fungi that may have the ability to promote growth or resist stress, which can be designed and selected specifically; (2) digging for and obtaining active antimicrobial strains with high efficiency and a broad spectrum for the biological control of plant diseases; (3) some active metabolites (matrine, quinorizidine alkaloids, etc.) produced from endophytic fungi that are the same or similar to host plants, which have extensive pharmacological activities and can be used in the development of new drugs for cancer and some chronic diseases; (4) it lays a foundation for introducing beneficial microorganisms into plants and promoting the healthy growth of plants, thus enriching the germplasm resources of *S. alopecuroides*.

## 5. Conclusions

In conclusion, a total of 751 OTUs were annotated from four organ *S. alopecuroides* samples in two sites, belonging to 9 phyla, 27 classes, 66 orders, 141 families, 245 genera, and 340 species. The dominant flora of the endophytic fungi of *S. alopecuroides* and the common flora of the four organs are unclassified Ascomycota, *Tricholoma*, *Apiotrichum*, *Alternaria*, *Aspergillus*, etc. The four organs of *S. alopecuroides* (root, stem, leaf, and seed) carried different fungal combinations and their dominant genera, endemic genera, and biomarkers are different, showing organ specificity or tissue preference, which is the most influential factor to explain the variation of the *S. alopecuroides* fungal community. The endophytic fungi of *S. alopecuroides* mainly had 15 ecological function groups: the saprotroph was absolutely dominant, the mixotrophic was second, and the symbiotroph was the least.

## Figures and Tables

**Table 1:** Diversity indexes of endophytic fungi of *S. alopecuroides* (mean ± SD).

Sample		Sequence	Ace	Simpson	Shannoneven	PD
site	H	58,139 ± 25,596	**90.55 ± 32.86 a**	0.31 ± 0.24	0.44 ± 0.16	26.03 ± 5.42
	B	53,271 ± 9435	**64.25 ± 23.22 b**	0.27 ± 0.11	0.49 ± 0.09	29.12 ± 6.96
organ	R	53,012 ± 3960	**106.24 ± 34.43 a**	0.23 ± 0.14	0.49 ± 0.11	28.59 ± 5.02
	St	53,832 ± 7679	**71.31 ± 28.21 a**	0.26 ± 0.23	0.51 ± 0.16	29.27 ± 5.98
	L	67,764 ± 33,229	**73.20 ± 28.86 a**	0.38 ± 0.26	0.43 ± 0.17	25.94 ± 7.16
	S	48,211 ± 15,229	**58.85 ± 10.10 b**	0.29 ± 0.07	0.43 ± 0.06	26.48 ± 7.83

Note: Different small letters indicate significant differences in diversity indexes (*p* < 0.05 in bold).

**Table 2:** Adonis pairwise comparisons of microbiota structure between sites and organs of *S. alopecuroides*.

Comparison		R^2^	*p*-Value
Sites comparison	H vs. B	0.0728	0.068
Organ comparison	Roots vs. Stems	0.3064	0.002
	Roots vs. Leaves	0.2935	0.002
	Roots vs. Seeds	0.3585	0.002
	Stems vs. Leaves	0.0625	0.723
	Stems vs. Seeds	0.1713	0.048
	Leaves vs. Seeds	0.2198	0.015

Note: The R^2^ value represents the degree of explanation for the grouping factors on the differences between the samples; a larger R^2^ indicates a higher degree of explanation of the differences by grouping; a *p*-value less than 0.05 indicates a high confidence level of this test.

**Table 3:** PERMANOVA analysis of endophytic fungal flora of *S. alopecuroides*.

Characteristics	SumsOfSqs	MeanSqs	F. Model	R^2^	*p*. Value	*P*. Adjust
organ	2.52902	0.84301	3.28379	0.33001	0.001	0.002
site	0.55774	0.55774	1.72685	0.07278	0.06	0.06

Note: The first column of the table represents the grouping factors, and the R^2^ value represents the degree of explanation of the grouping factors on the differences between the samples; a larger R^2^ indicates a higher degree of explanation of the differences by grouping; a *p*-value less than 0.05 indicates a high confidence level of this test.

**Table 4:** Common and unique genera of endophytic fungal fungi from different organs of *S. alopecuroides*.

Group Label	Number of Genera	Genus and Their Relative Abundance (>0.02)
Shared genera in 4 organs	33	unc. Ascomycota (18.42%), *Tricholoma* (15.31%), *Apiotrichum* (14.47%), *Alternaria* (12.35%), *Aspergillus* (9.65%), unc. Didymellaceae (7.96%), *Neocosmospora* (7.39%), unc. Fungi (2.09%), *Filobasidium* (2.06%), *Fusarium* (2.01%), others (8.29%)
Root unique genera	54	*Cadophora* (50.41%), *Setophoma* (14.10%), *Phaeomycocentrospora* (9.30%), *Leohumicola* (2.68%), *Ophiosimulans* (2.47%), unc. Sordariales (2.45%), unc. Ophiocordycipitaceae (2.44%), *Setophaeosphaeria* (2.41%), others (13.75%)
Stem unique genera	34	*Plenodomus* (38.25%), *Mrakia* (23.13%), *Sphaerosporella* (6.15%), *Phaeotremella* (4.74%), unc. Microascaceae (3.77%), unc. Lasiosphaeriaceae (2.65%), *Entrophospora* (2.39%), *Dominikia* (2.37%), *Clavulina* (2.13%), *Biatriospora* (2.01%), others (12.40%)
Leaf unique genera	30	*Dioszegia* (23.43%), *Sistotrema* (23.21%), *Thanatephorus* (11.10%), *Hawksworthiomyces* (8.41%), *Saitozyma* (5.61%), *Gonytrichum* (3.92%), unc. Xylariales (3.81%), *Humaria* (3.48%), *Hydnellum* (2.58%), unc. Metschnikowiaceae (2.24), unc. Mycosphaerellaceae (2.13%), others (10.09%)
Seed unique genera	22	*Papiliotrema* (69.88%), *Curvularia* (4.46%), *Stereum* (3.68%), unc. Pezizales (2.48%), others (19.50%)

## Data Availability

The ITS gene sequences of endophytes used in this manuscript were submitted to the NCBI and the Accession number is PRJNA832900.

## Supplementary Materials

The following supporting information can be downloaded at: https://www.mdpi.com/article/10.3390/microorganisms10112099/s1, Figure S1: Overall community composition of S. alopecuroides endophytic fungi at the genus level; Table S1: The alpha diversity indexes of each tissue type and two sites of *S. alopecuroides* endophytic fungi at the genus level (mean ± SD).

## References

[1] Gupta S., Chaturvedi P., Kulkarni M.G., Van Staden J. (2020). A critical review on exploiting the pharmaceutical potential of plant endophytic fungi. Biotechnol. Adv..

[2] An C., Ma S., Shi X., Xue W., Liu C., Ding H. (2020). Diversity and Antimicrobial Activity of Endophytic Fungi Isolated from *Chloranthus japonicus* Sieb in Qinling Mountains, China. Int. J. Mol. Sci..

[3] Hardoim P.R., van Overbeek L.S., Berg G., Pirttilä A.M., Compant S., Campisano A., Doring M., Sessitsch A. (2015). The Hidden World within Plants: Ecological and Evolutionary Considerations for Defining Functioning of Microbial Endophytes. Microbiol. Mol. Biol. Rev..

[4] Rodriguez R.J., White J.F., Arnold A.E., Redman R.S. (2009). Fungal endophytes: Diversity and functional roles. New Phytol..

[5] Vandenkoornhuyse P., Quaiser A., Duhamel M., Le Van A., Dufresne A. (2015). The importance of the microbiome of the plant holobiont. New Phytol..

[6] Simon J.C., Marchesi J.R., Mougel C., Selosse M.A. (2019). Host-microbiota interactions: From holobiont theory to analysis. Microbiome.

[7] Guo B., Wang Y., Sun X., Tang K. (2011). Bioactive natural products from endophytes: A review. Appl. Biochem. Microbiol..

[8] Gouda S., Das G., Sen S.K., Shin H.S., Patra J.K. (2016). Endophytes: A Treasure House of Bioactive Compounds of Medicinal Importance. Front. Microbiol..

[9] Vandana U.K., Rajkumari J., Singha L.P., Satish L., Alavilli H., Sudheer P., Chauhan S., Ratnala R., Satturu V., Mazumder P.B. (2021). The Endophytic Microbiome as a Hotspot of Synergistic Interactions, with Prospects of Plant Growth Promotion. Biology.

[10] Aly A.H., Debbab A., Proksch P. (2011). Fungal endophytes: Unique plant inhabitants with great promises. Appl. Microbiol. Biotechnol..

[11] Hartley S.E., Eschen R., Horwood J.M., Gange A.C., Hill E.M. (2015). Infection by a foliar endophyte elicits novel arabidopside-based plant defence reactions in its host, *Cirsium arvense*. New Phytol..

[12] Waller F., Achatz B., Baltruschat H., Fodor J., Becker K., Fischer M., Heier T., Hückelhoven R., Neumann C., Wettstein D.v. (2005). The endophytic fungus *Piriformospora indica* reprograms barley to salt-stress tolerance, disease resistance, and higher yield. Proc. Natl. Acad. Sci. USA.

[13] Wang Y., Zhou T., Li D., Zhang X., Yu W., Cai J., Wang G., Guo Q., Yang X., Cao F. (2019). The genetic diversity and population structure of *Sophora alopecuroides* (Faboideae) as determined by microsatellite markers developed from transcriptome. PLoS ONE.

[14] Wang R., Deng X., Gao Q., Wu X., Han L., Gao X., Zhao S., Chen W., Zhou R., Li Z. (2020). *Sophora alopecuroides* L.: An ethnopharmacological, phytochemical, and pharmacological review. J. Ethnopharmacol..

[15] Gu P., Hao L., Xu R., Hu M., Ma H. (2012). Diversity and antimicrobial activity of endophytic fungi in *Sophora alopecuroides* L. from Baijitan National Nature Reserve of Ningxia. J. Northwest AF Univ. Nat. Sci. Ed..

[16] Gao Y., Zhou X., Sun M., Lv M., Gu P. (2016). Diversity and Ecological Distribution of Endophytic Fungi of *Sophora alopecuroides* L. from Baijitan Nature Reserve of Ningxia. Acta Agrestia Sin..

[17] Yan K., Pei Z., Meng L., Zheng Y., Wang L., Feng R., Li Q., Liu Y., Zhao X., Wei Q. (2022). Determination of Community Structure and Diversity of Seed-Vectored Endophytic Fungi in *Alpinia zerumbet*. Front. Microbiol..

[18] Han S., Wang Y., Li Y., Shi K. (2021). Investigation of bacterial diversity in *Cajanus cajan*-planted gangue soil via high-throughput sequencing. Bioengineered.

[19] Glynou K., Nam B., Thines M., Macia-Vicente J.G. (2018). Facultative root-colonizing fungi dominate endophytic assemblages in roots of nonmycorrhizal *Microthlaspi* species. New Phytol..

[20] Ren F., Dong W., Yan D.H. (2019). Organs, Cultivars, Soil, and Fruit Properties Affect Structure of Endophytic Mycobiota of Pinggu Peach Trees. Microorganisms.

[21] Xue F., Liu T. (2021). DNA sequence and community structure diversity of multi-year soil fungi in Grape of Xinjiang. Sci. Rep..

[22] Zhang P., Guan P., Hao C., Yang J., Xie Z., Wu D. (2022). Changes in assembly processes of soil microbial communities in forest-to-cropland conversion in Changbai Mountains, northeastern China. Sci. Total Environ..

[23] Chen Y., Wu B., Zhang C., Fan Z., Chen Y., Xin B., Xie Q. (2020). Current Progression: Application of High-Throughput Sequencing Technique in Space Microbiology. Biomed. Res. Int..

[24] Rego S.M., Snyder M.P. (2019). High Throughput Sequencing and Assessing Disease Risk. Cold Spring Harb. Perspect. Med..

[25] Chan A.W., Naphtali J., Schellhorn H.E. (2019). High-throughput DNA sequencing technologies for water and wastewater analysis. Sci. Prog..

[26] Latz M.A.C., Kerrn M.H., Sorensen H., Collinge D.B., Jensen B., Brown J.K.M., Madsen A.M., Jorgensen H.J.L. (2021). Succession of the fungal endophytic microbiome of wheat is dependent on tissue-specific interactions between host genotype and environment. Sci. Total Environ..

[27] Abdelfattah A., Cacciola S.O., Mosca S., Zappia R., Schena L. (2017). Analysis of the Fungal Diversity in Citrus Leaves with Greasy Spot Disease Symptoms. Microb. Ecol..

[28] Alibrandi P., Schnell S., Perotto S., Cardinale M. (2020). Diversity and Structure of the Endophytic Bacterial Communities Associated with Three Terrestrial Orchid Species as Revealed by 16S rRNA Gene Metabarcoding. Front. Microbiol..

[29] Hou Q.Z., Chen D.W., Wang Y.P., Ehmet N., Ma J., Sun K. (2022). Analysis of endophyte diversity of two *Gentiana* plants species and the association with secondary metabolite. BMC Microbiol..

[30] Cui L., Noushahi H.A., Zhang Y., Liu J., Cosoveanu A., Liu Y., Yan L., Zhang J., Shu S. (2021). Endophytic Fungal Community of *Huperzia serrata*: Diversity and Relevance to the Production of Huperzine A by the Plant Host. Molecules.

[31] Liang Y., Wei G., Ning K., Zhang G., Liu Y., Dong L., Chen S. (2021). Contents of lobetyolin, syringin, and atractylolide III in *Codonopsis pilosula* are related to dynamic changes of endophytes under drought stress. Chin. Med..

[32] Ju M., Zhang Q., Wang R., Yan S., Li Z., Li P., Gu P. (2022). Correlation in endophytic fungi community diversity and bioactive compounds of *Sophora alopecuroides*. Front. Microbiol..

[33] Houlden A., Timms-Wilson T.M., Day M.J., Bailey M.J. (2008). Influence of plant developmental stage on microbial community structure and activity in the rhizosphere of three field crops. FEMS Microbiol. Ecol..

[34] Li Y., He X., Yuan H., Lv G. (2022). Differed Growth Stage Dynamics of Root-Associated Bacterial and Fungal Community Structure Associated with Halophytic Plant *Lycium ruthenicum*. Microorganisms.

[35] Ajilogba C.F., Olanrewaju O.S., Babalola O.O. (2022). Plant Growth Stage Drives the Temporal and Spatial Dynamics of the Bacterial Microbiome in the Rhizosphere of *Vigna subterranea*. Front. Microbiol..

[36] Puri S.C., Nazir A., Chawla R., Arora R., Riyaz-ul-Hasan S., Amna T., Ahmed B., Verma V., Singh S., Sagar R. (2006). The endophytic fungus *Trametes hirsuta* as a novel alternative source of podophyllotoxin and related aryl tetralin lignans. J. Biotechnol..

[37] Adams R.I., Miletto M., Taylor J.W., Bruns T.D. (2013). Dispersal in microbes: Fungi in indoor air are dominated by outdoor air and show dispersal limitation at short distances. ISME J..

[38] Martin M. (2011). Cutadapt removes adapter sequences from high-throughput sequencing reads. EMBnet. J..

[39] Duan Y., Lian J., Wang L., Wang X., Luo Y., Wang W., Wu F., Zhao J., Ding Y., Ma J. (2021). Variation in Soil Microbial Communities Along an Elevational Gradient in Alpine Meadows of the Qilian Mountains, China. Front. Microbiol..

[40] Nilsson R.H., Anslan S., Bahram M., Wurzbacher C., Baldrian P., Tedersoo L. (2019). Mycobiome diversity: High-throughput sequencing and identification of fungi. Nat. Rev. Microbiol..

[41] Magoč T., Salzberg S.L. (2011). FLASH: Fast length adjustment of short reads to improve genome assemblies. Bioinformatics.

[42] Caporaso J.G., Kuczynski J., Stombaugh J., Bittinger K., Bushman F.D., Costello E.K., Fierer N., Peña A.G., Goodrich J.K., Gordon J.I. (2010). QIIME allows analysis of high-throughput community sequencing data. Nat. Methods.

[43] Bokulich N.A., Subramanian S., Faith J.J., Gevers D., Gordon J.I., Knight R., Mills D.A., Caporaso J.G. (2013). Quality-filtering vastly improves diversity estimates from Illumina amplicon sequencing. Nat. Methods.

[44] Alawneh L., Debbabi M., Hassaine F., Jarraya Y., Shahi P., Soeanu A. Towards a unified paradigm for verification and validation of systems engineering design models. Proceedings of the IASTED, Innsbruck(AT).

[45] Edgar R.C., Haas B.J., Clemente J.C., Quince C., Knight R. (2011). UCHIME improves sensitivity and speed of chimera detection. Bioinformatics.

[46] Edgar R.C. (2013). UPARSE: Highly accurate OTU sequences from microbial amplicon reads. Nat. Methods.

[47] Schloss P.D., Westcott S.L., Ryabin T., Hall J.R., Hartmann M., Hollister E.B., Lesniewski R.A., Oakley B.B., Parks D.H., Robinson C.J. (2009). Introducing mothur: Open-Source, Platform-Independent, Community-Supported Software for Describing and Comparing Microbial Communities. Appl. Environ. Microbiol..

[48] Vázquez-Baeza Y., Pirrung M., Gonzalez A., Knight R. (2013). EMPeror: A tool for visualizing high-throughput microbial community data. GigaScience.

[49] Siddique A.B., Biella P., Unterseher M., Albrectsen B.R. (2021). Mycobiomes of Young Beech Trees Are Distinguished by Organ Rather than by Habitat, and Community Analyses Suggest Competitive Interactions among Twig Fungi. Front. Microbiol..

[50] Anderson M.J. (2001). A new method for non-parametric multivariate analysis of variance. Austral Ecol..

[51] Wu Q., Wei D., Dong L., Liu Y., Ren C., Liu Q., Chen C., Chen J., Pei J. (2019). Variation in the microbial community contributes to the improvement of the main active compounds of *Magnolia officinalis* Rehd. et Wils in the process of sweating. Chin. Med..

[52] Nguyen N.H., Song Z., Bates S.T., Branco S., Tedersoo L., Menke J., Schilling J.S., Ecology P.G.K.A.J.F. (2016). FUNGuild: An open annotation tool for parsing fungal community datasets by ecological guild. Fungal Ecol..

[53] Segata N., Izard J., Waldron L., Gevers D., Miropolsky L., Garrett W.S., Huttenhower C. (2011). Metagenomic biomarker discovery and explanation. Genome Biol..

[54] Yu Y., He S., Zhao Q. (2013). Isolation and Identification of Matrine-Producing Fungal Endophytes from *Sophora alopecuroides* in Ningxia. Sci. Agric. Sin..

[55] Gamboa M.A., Laureano S., Bayman P. (2002). Measuring diversity of endophytic fungi in leaf fragments: Does size matter?. Mycopathologia.

[56] Wijayawardene N.N., Bahram M., Sanchez-Castro I., Dai D.Q., Ariyawansa K., Jayalal U., Suwannarach N., Tedersoo L. (2021). Current Insight into Culture-Dependent and Culture-Independent Methods in Discovering Ascomycetous Taxa. J. Fungi.

[57] Edwards J., Johnson C., Santos-Medellin C., Lurie E., Podishetty N.K., Bhatnagar S., Eisen J.A., Sundaresan V. (2015). Structure, variation, and assembly of the root-associated microbiomes of rice. Proc. Natl. Acad. Sci. USA.

[58] Berg G., Rybakova D., Grube M., Köberl M. (2016). The plant microbiome explored: Implications for experimental botany. J. Exp. Bot..

[59] Küngas K., Bahram M., Põldmaa K. (2020). Host tree organ is the primary driver of endophytic fungal community structure in a hemiboreal forest. FEMS Microbiol. Ecol..

[60] Müller D.B., Vogel C., Bai Y., Vorholt J.A. (2016). The Plant Microbiota: Systems-Level Insights and Perspectives. Annu. Rev. Genet..

[61] Sun J., Guo L., Zang W., Ping W., Chi D. (2008). Diversity and ecological distribution of endophytic fungi associated with medicinal plants. Sci. China C Life Sci..

[62] Su Y., Guo L., Hyde K.D. (2010). Response of endophytic fungi of *Stipa grandis* to experimental plant function group removal in Inner Mongolia steppe, China. Fungal Divers..

[63] Chen M., Chen J., Liu J., Wu M., Yan Q., Li P., Huang L., Xiao X. (2021). Diversity analysis of rhizosphere soil fungi and endophytic fungi in *Ampelocalamus luodianensis*. Acta Ecol. Sin..

[64] Yao X., Kang Q., Xiong S., Li F., Wang Y., Lin S., Bai L., Ma W., Deng Z. (2014). Isolation and identification of endophytic actinomycetes from the seeds of *Camptotheca acuminata* Decne. and isolation of antimicrobial substances from those endophytic actinomycetes. Microbiol. China.

[65] Root R. (1967). The niche exploitation pattern of the blue-grey gnatcatcher. Ecol. Monogr..

[66] Zhou J., Xie T., Liu J., Lan L., Xu Y., Liu Z., Ai Y., Zhang Q. (2021). Community structure and biological function of the root symbiotic fungi of wild *Cymbidium ensifolium*. Acta Microbiol. Sin..

[67] Zhou J., Wang M., Yu L., Liu J., Zou X. (2019). The structure and function of endophytic fungal community in tobacco root. Mycosystema.

[68] Arnold A.E., Lutzoni F. (2007). Diversity and host range of foliar fungal endophytes: Are tropical leaves biodiversity hotspots?. Ecology.

[69] Baarlen P.V., Belkum A.V., Summerbell R.C., Crous P.W., Thomma B.P.H.J. (2007). Molecular mechanisms of pathogenicity: How do pathogenic microorganisms develop cross-kingdom host jumps?. FEMS Microbiol. Rev..

[70] Nagy N.E., Fossdal C.G. (2013). Host responses in Norway spruce roots induced to the pathogen *Ceratocystis polonica* are evaded or suppressed by the ectomycorrhizal fungus *Laccaria bicolor*. Plant Biol..

[71] Grau O., Geml J., Pérez-Haase A., Ninot J.M., Semenova-Nelsen T.A., Peñuelas J. (2017). Abrupt changes in the composition and function of fungal communities along an environmental gradient in the high Arctic. Mol. Ecol..

[72] Chu H., Wang C., Wang H., Chen H., Tang M. (2016). Pine wilt disease alters soil properties and root-associated fungal communities in *Pinus tabulaeformis* forest. Plant Soil.

[73] Millberg H., Boberg J., Stenlid J. (2015). Changes in fungal community of Scots pine (*Pinus sylvestris*) needles along a latitudinal gradient in Sweden. Fungal Ecol..

[74] Semchenko M., Leff J.W., Lozano Y.M., Saar S., Davison J., Wilkinson A., Jackson B.G., Pritchard W.J., Long J.R.D., Oakley S. (2018). Fungal diversity regulates plant-soil feedbacks in temperate grassland. Sci. Adv..

[75] Wang H., Guo S., Huang M., Thorsten L.H., Wei J. (2010). Ascomycota has a faster evolutionary rate and higher species diversity than Basidiomycota. Sci. China Life Sci..

[76] Elkady W.M., Raafat M.M., Abdel-Aziz M.M., Al-Huqail A.A., Ashour M.L., Fathallah N. (2022). Endophytic Fungus from Opuntia ficus-indica: A Source of Potential Bioactive Antimicrobial Compounds against Multidrug-Resistant Bacteria. Plants.

[77] Morales-Sanchez V., Diaz C.E., Trujillo E., Olmeda S.A., Valcarcel F., Munoz R., Andres M.F., Gonzalez-Coloma A. (2021). Bioactive Metabolites from the Endophytic Fungus *Aspergillus* sp. SPH2. J. Fungi.

[78] Xiao J., Zhang Q., Gao Y.Q., Shi X.W., Gao J.M. (2014). Antifungal and antibacterial metabolites from an endophytic *Aspergillus* sp. associated with *Melia azedarach*. Nat. Prod. Res..

[79] Khan A.L., Hamayun M., Kim Y.-H., Kang S.-M., Lee J.-H., Lee I.-J. (2011). Gibberellins producing endophytic *Aspergillus fumigatus* sp. LH02 influenced endogenous phytohormonal levels, isoflavonoids production and plant growth in salinity stress. Process Biochem..

[80] Mehmood A., Hussain A., Irshad M., Hamayun M., Iqbal A., Khan N. (2018). In vitro production of IAA by endophytic fungus *Aspergillus awamori* and its growth promoting activities in Zea mays. Symbiosis.

[81] Kwon Y.-J., Sohn M.-J., Zheng C.-J., Kim W.-G. (2007). Fumimycin: A Peptide Deformylase Inhibitor with an Unusual Skeleton Produced by *Aspergillus fumisynnematus*. Org. Lett..

[82] Woudenberg J.H., Groenewald J.Z., Binder M., Crous P.W. (2013). *Alternaria* redefined. Stud. Mycol..

[83] Wang Y., Liu H.X., Chen Y.C., Sun Z.H., Li H.H., Li S.N., Yan M.L., Zhang W.M. (2017). Two New Metabolites from the Endophytic Fungus *Alternaria* sp. A744 Derived from *Morinda officinalis*. Molecules.

[84] Xiang L., Gong S., Yang L., Hao J., Xue M., Zeng F., Zhang X., Shi W., Wang H., Yu D. (2016). Biocontrol potential of endophytic fungi in medicinal plants from Wuhan Botanical Garden in China. Biol. Control.

[85] Sun M., Zhang Q., Hu L., Li W., Yan S., Lv M., Gu P. (2018). Real-time fluorescent quantitative PCR detection of key enzyme genes expression of alkaloid biosynthesis promoted by endophytic fungal elicitor in *Sophora alopecuroides*. Chin. Tradit. Herb. Drugs.

