# Supplementary material for: The Endophytic Fungi Diversity, Community Structure, and Ecological Function Prediction of Sophora alopecuroides in Ningxia, China
Source: Microorganisms. 2022 Oct 22;10(11):2099. doi: 10.3390/microorganisms10112099 (PMC9695620; doi:10.3390/microorganisms10112099)
Supplement: Supplementary file 1 [file microorganisms-10-02099-s001.zip › microorganisms-1903285-supplementary.pdf]

Supplementary materials

Supplementary Table.S1 The Alpha-Diversity indexes of each tissue type and two sites of *S. alopecuroides* endophytic fungi at the genus level (mean ± SD).

Sample		sequence	Ace	Simpson	Shannoneven	PD
	HR	50437±3080	114.47±51.46	0.27±0.19	0.45±0.13	26.31±4.96
	HSt	51361±6789	89.23±31.91	0.32±0.35	0.45±0.23	28.14±6.87
H	HL	79608±46329	94.03±18.67	0.36±0.40	0.42±0.25	25.77±7.34
	HS	51149±21880	64.48±5.02	0.29±0.04	0.43±0.02	23.88±4.70
	mean	58139±25596	90.55±32.86	0.31±0.24	0.44±0.16	26.02±5.42
	BR	55586±3137	98.01±10.54	0.18±0.07	0.53±0.09	30.88±4.77
	BSt	56303±9111	53.4±2.93	0.20±0.04	0.57±0.06	30.39±6.19
B	BL	55921±13903	52.37±20.78	0.39±0.12	0.43±0.07	26.11±8.62
	BS	45273±8671	53.22±11.61	0.30±0.10	0.44±0.10	29.09±10.52
	mean	53271±9435	64.25±23.22	0.27±0.11	0.49±0.09	29.12±6.96

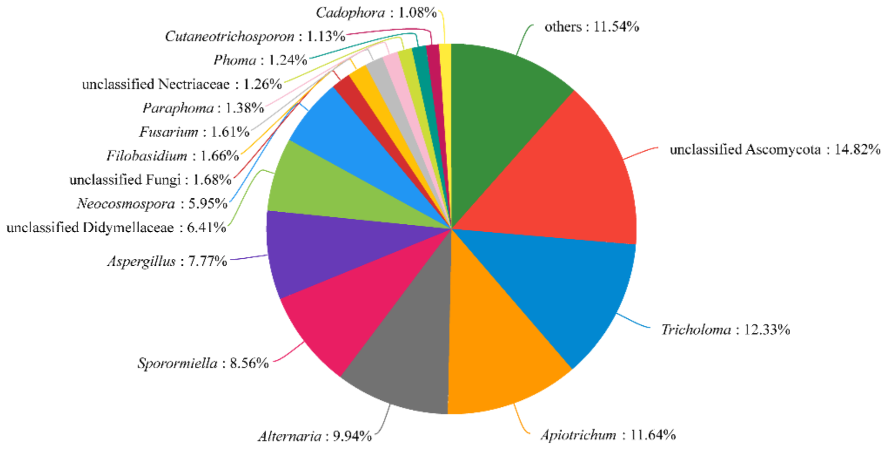

Supplementary Figure.S1 Overall community composition of *S. alopecuroides* endophytic fungi at the genus level.
